# Pharmacokinetics of Isavuconazole During Extracorporeal Membrane Oxygenation Support in Critically Ill Patients: A Case Series

**DOI:** 10.3390/antibiotics14060600

**Published:** 2025-06-12

**Authors:** Laura Doménech-Moral, Sonia García-García, Alba Pau-Parra, Manuel Sosa, Adrian Puertas Sanjuan, Camilo Bonilla, Elisabeth Gallart, Laura Castellote, Patricia Faixó, Jessica Guevara, Albert Vilanova, María Martínez-Pla, Aldair Conto, Xavier Nuvials, Pilar Lalueza, Ricard Ferrer, Maria Queralt Gorgas, Jordi Riera

**Affiliations:** 1 Pharmacy Department, Vall d’Hebron University Hospital, Basic, Translational and Clinical Pharmacy Research Group, Vall d’Hebron Research Institute, 08035 Barcelona, Spain; 2 Critical Care Department, Vall d’Hebron University Hospital, SODIR, Vall d’Hebron Research Institute, 08035 Barcelona, Spainjordi.rieradelbrio@vallhebron.cat (J.R.); 3 Department of Clinical Biochemistry, Vall d’Hebron University Hospital, 08035 Barcelona, Spain

**Keywords:** Isavuconazole, pharmacokinetics, antifungal therapy, critical care, V-V ECMO, therapeutic drug monitoring, drug sequestering

## Abstract

Background/Objectives: Extracorporeal membrane oxygenation (ECMO) is increasingly used in critically ill patients, but may significantly alter the pharmacokinetics (PK) of antifungals. Data on plasma concentrations of Isavuconazole (IsaPlasm) in ECMO patients are limited. Our objective is to evaluate Isavuconazole exposure and variability in critically ill COVID-19 patients receiving ECMO. Methods: We conducted a pharmacokinetic analysis of Isavuconazole in critically ill patients receiving Veno-Venous ECMO for respiratory support. Plasma concentrations were measured using therapeutic drug monitoring (TDM) at multiple time points, including sampling before and after the membrane oxygenator. PK parameters—Area Under Curve (AUC_0–24_), Minimum Plasma Concentration (Cmin), Elimination Half-Life (T_1/2_), volume of distribution (Vd), and clearance (CL)—were estimated and compared with published data in non-ECMO populations. Results: Five patients were included. The median AUC_0–24_ was 227.3 µg·h/mL (IQR 182.4–311.35), higher than reported in non-ECMO patients. The median Vd was 761 L (727–832), suggesting extensive peripheral distribution and potential drug sequestration in the ECMO circuit. CL was increased (1.6 L/h, IQR 1.5–3.4). Two patients with recently replaced ECMO circuits exhibited significant drug loss across the membrane. Obesity and hypoalbuminemia were identified as factors associated with altered drug exposure. Conclusions: Isavuconazole pharmacokinetics show marked variability in critically ill ECMO patients. Increased AUC and Vd, along with reduced clearance, highlight the need for individualized dosing.

## 1. Introduction

Isavuconazole is a broad-spectrum triazole antifungal approved for the treatment of invasive aspergillosis and mucormycosis [1]. Its favorable pharmacological profile—including high oral bioavailability, lack of cyclodextrin in the intravenous formulation, predictable pharmacokinetics, and a lower potential for hepatotoxicity and drug–drug interactions—makes it particularly suitable for use in critically ill patients [2,3]. 

Among the emerging indications, COVID-19-associated pulmonary aspergillosis (CAPA) has gained increasing attention as a life-threatening superinfection in patients with severe SARS-CoV-2 pneumonia. CAPA is associated with epithelial damage, immune dysregulation, and the use of immunomodulatory therapies such as corticosteroids and IL-6 inhibitors [4,5]. Early antifungal treatment is essential, and Isavuconazole is considered a first-line agent, alongside voriconazole [6].

Although therapeutic drug monitoring (TDM) is not routinely recommended for Isavuconazole due to limited data from clinical trials such as the SECURE study—where most patients achieved plasma concentrations above 1 mg/L without TDM [7]—emerging evidence supports its potential utility in specific populations.

Real-world studies have shown that critically ill patients, particularly those in the Intensive Care Unit (ICU) or receiving ECMO, often present lower than expected Isavuconazole plasma concentrations and greater pharmacokinetic variability [8,9,10,11]. Pérez et al. [12] observed that standard dosing frequently failed to achieve target trough concentrations ≥2 mg/L within 72 h in critically ill patients with CAPA, supporting the use of individualized dosing strategies. 

Moreover, Furfaro et al. [13] found that TDM-guided dose adjustments were often necessary to avoid both subtherapeutic concentrations and toxicity. In their study, Isavuconazole plasma concentrations above 4.87–5.86 mg/L were associated with a higher risk of adverse events, including drug-induced liver injury, diarrhea, and nausea—all of which resolved after dose reduction. Therefore, while not routinely indicated, TDM should be considered in critically ill patients and others at risk of altered pharmacokinetics, in line with the increasing clinical evidence. 

During the COVID-19 pandemic, extracorporeal membrane oxygenation (ECMO) became a common intervention in patients with refractory acute respiratory distress syndrome (ARDS). Notably, post-first wave data indicated a high incidence of CAPA in ECMO-supported patients [14,15]. However, ECMO circuits can significantly alter drug pharmacokinetics due to sequestration within the circuit, changes in volume of distribution, and altered clearance [16]—particularly for drugs with high lipophilicity and plasma protein binding such as Isavuconazole.

The objectives of this study are to characterize the relationship between administered Isavuconazole doses and plasma Isavuconazole concentrations (IsaPlasm) in critically ill patients receiving ECMO support, as well as to assess potential differences in IsaPlasm at different points within the ECMO circuit to investigate drug sequestration phenomena. Additionally, the study aims to evaluate the clinical effectiveness and safety of Isavuconazole therapy in this population, with a focus on treatment outcomes and the occurrence of adverse events. 

## 2. Results

### 2.1. Cohort Description

Five critically ill patients with ARDSsecondary to COVID-19 who required veno-venous ECMO (V-V ECMO) support were included in the study. The median age was 58 years (range: 44–65), and two patients (33.3%) were female. The median body mass index (BMI) was 26.4 Kg/m^2^ (range: 22.6–36.4). None of the patients had chronic renal impairment or hepatic failure at the time of ECMO initiation. 

Isavuconazole was initiated in four patients diagnosed with probable COVID-19-associatedCAPA according to the ECMM/ISHAM 2020 consensus criteria [6]. In one additional patient, antifungal therapy was started empirically due to a clinical suspicion of possible CAPA, but was subsequently discontinued after a negative galactomannan assay and the absence of microbiological confirmation. 

The median duration of Isavuconazole treatment was 71 days (IQR: 7–92). No patients discontinued Isavuconazole due to drug-related adverse events. 

Survival outcomes were as follows: 80% (4/5) survived ECMO therapy, 60% (3/5) were discharged from the ICU, and 60% (3/5) remained alive 90 days after ICU discharge. 

Among the four patients treated for probable CAPA, two died due to causes unrelated to fungal infection, and two had negative respiratory control cultures. The patient who received empirical antifungal therapy was later ruled out for fungal infection and Isavuconazole was discontinued. 

### 2.2. Patient Characteristics and Pharmacokinetic Parameters

Table 1 summarizes individual patient characteristics, treatment details, laboratory values, and Isavuconazole exposure (AUC values). None of the patients required renal replacement therapy. Additionally, a review of concomitant medications revealed no evidence of clinically significant pharmacokinetic drug–drug interactions that could have affected the disposition of the study drug.

Total daily Isavuconazole exposure (AUC_0–24_) ranged from 135.7 to 395.4 µg·h/mL across the cohort. Isavuconazole exposure appeared higher in patients with elevated bilirubin or lower albumin levels, although the small sample size limited formal statistical analysis. 

### 2.3. ECMO Circuit Effect on Isavuconazole Concentration and Pharmacokinetics

AUC values were determined after achieving steady-state plasma concentrations (median: 5 days, IQR: 4–9). The impact of the ECMO circuit on Isavuconazole pharmacokinetics was assessed by comparing drug concentrations pre- and post-oxygenator on C0 and C1 points. 

The Isavuconazole plasma concentration–time profiles from arterial sampling points and across the ECMO circuit are summarized in Table 2.

In two patients (P3 and P5) who had recently undergone ECMO circuit replacement (within the previous 3 days), a marked difference between pre- and post-oxygenator Isavuconazole concentrations was observed, suggesting possible drug sequestration by the new oxygenator membrane or other circuit components.

Table 3 provides a comparative overview of key PK parameters, including AUC, Cmin, t1/2, kel, Vd and CL, of the cases presented in this article, alongside previously published data in non-ECMO settings and standard prescribing information. This comparative analysis highlights differences in drug exposure and pharmacokinetic behavior associated with ECMO support. 

**Figure 1:**
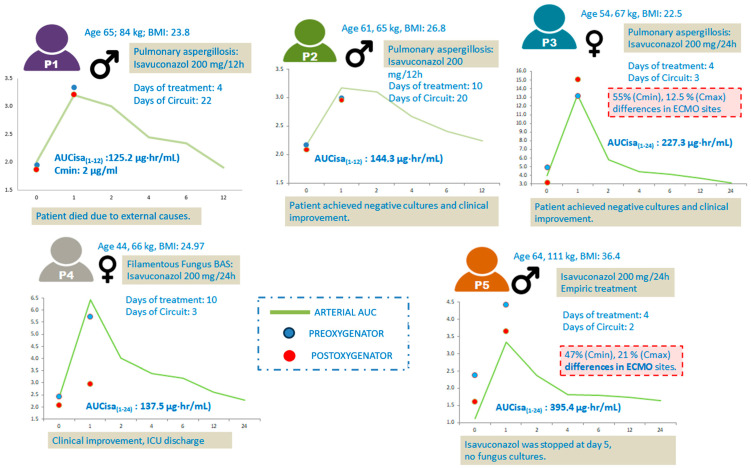
Pharmacokinetic profiles of five critically ill patients undergoing ECMO while receiving Isavuconazole. Each panel includes patient demographics, treatment regimen, days on ECMO, pharmacokinetic parameters (AUC₍_1–12_₎ or AUC₍_1–24_₎ and Cmin), and clinical evolution. Arterial Isavuconazole levels are represented by green curves. Red and blue dots indicate post- and pre-oxygenator sample concentrations, respectively.

A visual summary of individual pharmacokinetic profiles, patient characteristics, treatment duration, and clinical outcomes is presented in Figure 1. The figure highlights relevant interindividual differences in Isavuconazole exposure (AUC, Cmin), circuit-related variability (pre-/post-oxygenator differences), and the corresponding clinical course. Notably, P5 showed the highest AUC (395.4 µg·h/mL) with the lowest Cmin (1.32 µg/mL), and relevant differences (up to 47%) between ECMO sampling sites, whereas P3 achieved the highest Cmin (3.96 µg/mL) and negative cultures within 4 days.

Figure 1 shows a summary of individual pharmacokinetic profiles.

### 2.4. Clinical Features of the Cohort

Patient 1 (65-year-old male) developed CAPA confirmed by *Aspergillus fumigatus* isolation in bronchoalveolar lavage (BAL) fluid and a positive BAL galactomannan (GM) index of 0.640. Despite antifungal therapy, he died from disease progression.

Patient 2 (61-year-old male) was diagnosed with CAPA based on the isolation of *Aspergillus flavus* in BAL and bronchial aspirates, and a BAL GM index of 0.7. Serum GM was negative. Chest CT revealed multiple nodules and areas of consolidation suggestive of invasive aspergillosis. He improved and was discharged from the ICU. Two years later, he underwent bilateral lung transplantation for post-COVID pulmonary fibrosis.

Patient 3 (57-year-old female) had a history of multiple sclerosis treated with rituximab and developed CAPA caused by *Aspergillus terreus*. BAL and bronchial aspirate cultures were positive, with a BAL GM index of 1.2 and positive serum GM. Imaging showed features consistent with invasive pulmonary aspergillosis. She responded to treatment and was discharged from the ICU.

Patient 4 (44-year-old female) had a history of bilateral lung transplantation under chronic immunosuppression and was diagnosed with CAPA based on positive BAL and serum GM and *Aspergillus* spp. isolation from respiratory samples. Despite antifungal therapy, she died from multiple infectious complications.

Patient 5 (64-year-old male) received empirical antifungal therapy due to clinical deterioration and high risk of fungal infection in the context of COVID-19 and *Pseudomonas aeruginosa* coinfection. Isavuconazole was initiated but discontinued early after negative fungal cultures and serum biomarkers. He improved and was successfully discharged from the ICU.

Chest CT findings in patients with probable CAPA included multiple pulmonary nodules with surrounding ground-glass opacities (halo sign), cavitary lesions, and areas of consolidation. In two cases, the air crescent sign was observed during recovery. All four CAPA patients had received corticosteroids and/or immunomodulatory therapy, supporting the diagnosis of invasive fungal infection.

Isavuconazole treatment was generally well tolerated. One patient experienced mild diarrhea, and another developed isolated cholestasis. In both cases, these adverse events were not conclusively attributed to Isavuconazole, as concomitant medications used in critical care may have contributed.

## 3. Discussion

This prospective observational study provides insights into the pharmacokinetics of Isavuconazole in critically ill patients with ARDS undergoing VV-ECMO support. To our knowledge, this is one of the few studies to provide real-time measurements of Isavuconazole plasma concentrations across the ECMO circuit, including pre- and post-membrane sampling, allowing for a comprehensive assessment of drug exposure (AUC) and circuit-related sequestration.

ECMO is increasingly utilized as a life-saving intervention in cases of refractory respiratory or cardiac failure. However, its impact on drugPK is complex and often unpredictable. The extracorporeal circuit can significantly alter the PK parameters of antimicrobial agents, potentially leading to subtherapeutic plasma concentrations and therapeutic failure [18]. Despite its clinical relevance, data on the pharmacokinetics of Isavuconazole in ECMO are scarce, primarily derived from case reports or small series [12,19,20,21,22,23], making it challenging to establish appropriate dosing guidelines.

In our cohort of five critically ill patients, we observed substantial interindividual variability in Isavuconazole exposure, with AUC0–24 values ranging from 137.5 to 395.4 µg·h/mL, with a median of 250.4 (182–288) µg·h/mL. Notably, the median AUC in our patients was higher than reported in non-ECMO populations, with values of 89.6 µg·h/mL in Desai et al. [24] and 105.3 ± 55.9 µg·h/mL in Kovanda et al. [25]. The Desai population corresponds to patients from the SECURE trial, which included non-critically ill individuals with invasive aspergillosis and without ECMO support—features that contrast markedly with the clinical characteristics of our cohort. Multiple factors may account for this increase, including heightened systemic inflammation, older age, and potential reductions in drug clearance associated with hepatic impairment [26]. In our cohort, no patients received CYP3A inducers or inhibitors, yet interindividual variability persisted, underscoring the unpredictable nature of Isavuconazole kinetics in critically ill patients with ECMO support.

Hypoalbuminemia and liver dysfunction have been previously identified as key determinants of Isavuconazole exposure [26,27]. Bolcato et al. [28] demonstrated that AST and albumin levels were independently associated with trough concentrations, with inter- and intra-individual variability of 41.5% and 30.7%, respectively. In our study, we observed considerable variability in AUC and trough concentrations, likely driven by similar mechanisms. Given that Isavuconazole is >99% plasma protein-bound, hypoalbuminemia may enhance the unbound (pharmacologically active) fraction, thereby increasing tissue distribution and potentially accelerating clearance.

Regarding Vd, our patients demonstrated a higher median Vd (599 L) compared to non-ECMO populations. For instance, Desai’s model estimated a Vd of 260 L [24], while Kovanda et al. reported 354 L [25]. The observed expansion in Vd may reflect altered capillary permeability, fluid shifts, and sequestration within the ECMO circuit. Additionally, the median elimination half-life (30 h) was substantially shorter than expected for Isavuconazole in healthy individuals (100–130 h) [25,29], suggesting enhanced drug clearance potentially driven by pathophysiological changes in critically ill patients and ECMO-related drug loss. Interestingly, the discrepancy between the increased Vd and the shortened half-life underscores that the augmented clearance likely outweighs the distributional expansion, given that elimination half-life is determined by both Vd and clearance.

Obesity may further complicate Isavuconazole pharmacokinetics, as increased adipose tissue can significantly affect the volume of distribution and clearance. In our cohort, one patient with a BMI of 36.4 kg/m^2^ demonstrated a prolonged elimination half-life, likely due to increased Vd associated with high adipose tissue content and protein binding. Prior studies [11,30] have indicated that patients with a BMI >25 may exhibit lower plasma concentrations, potentially necessitating dose adjustments. In such cases, early therapeutic drug monitoring is crucial to balance efficacy and avoid toxicity [8,31].

Interestingly, we observed a significant post-membrane drop in Isavuconazole concentrations in two patients who had undergone recent ECMO circuit changes, suggesting potential early-phase drug sequestration due to unsaturated binding sites in new circuit components. This finding aligns with previous reports of antifungal drug loss within ECMO circuits, particularly during the early phase of circuit initiation or replacement [21,22]. Zhao et al. [22] documented similar findings with liposomal amphotericin B and Isavuconazole, indicating that ECMO circuit age and composition may play critical roles in drug sequestration patterns.

TDMmay be instrumental in optimizing Isavuconazole dosing in ECMO-supported patients. Several studies have suggested that higher or adaptive dosing strategies may be required to achieve therapeutic concentrations in this population [11,30]. Notably, Hatzl et al. [32] reported that an initial loading dose of 400 mg achieved significantly higher plasma concentrations compared to standard dosing, without increased toxicity. Similarly, Jansen et al. [33] emphasized the limitations of standard dosing in critically ill patients and advocated for TDM-guided dose adjustments to prevent subtherapeutic exposure.

Despite these findings, our study has several limitations. First, the small sample size limits the generalizability of our results and precludes robust statistical analysis. Additionally, we only collected two PK sampling points to minimize blood draws, which may reduce accuracy. Lastly, only total drug levels were measured, preventing analysis of the free drug fraction—a critical consideration in hypoalbuminemic patients.

## 4. Materials and Methods

### 4.1. Study Design and Population

This was a prospective observational cohort study conducted in adult patients (≥18 years) who received ECMO support and were treated with Isavuconazole either for prophylaxis or fungal infections. Patients were admitted to the ICU of a tertiary academic medical center between September 2021 and March 2023. The study protocol was approved by the Institutional Review Board and written or witnessed verbal informed consent was obtained from all patients or their legally authorized representatives prior to study enrolment. 

### 4.2. Isavuconazole Treatment

Based on the recommendations from local drug advisory committees, Isavuconazole was prescribed in patients meeting the criteria for possible, probable, or proven invasive aspergillosis using the Blot criteria for invasive aspergillosis in critically ill patients. The treatment regimen consisted of a loading dose of Isavuconazole 200 mg every 8 h for 48 h, followed by a maintenance dose of 200 mg once daily, as specified in the summary of product characteristics [18]. In selected cases, an empirically increased maintenance dose of 200 mg twice daily was administered based on published literature [29]. All intravenous infusions were administered over 60 min, according to manufacturer recommendations [18]. 

A therapeutic target for Isavuconazole trough plasma concentrations ranging from 2.5 to 5.0 µg/mL was considered appropriate for both prophylaxis and the treatment of invasive fungal infections. The selected range is based on the mean trough levels observed in the SECURE trial (mean on day 7: 2.6 ± 1.0 µg/mL), and the upper limit was determined considering clinical evidence suggesting a higher incidence of adverse events, particularly gastrointestinal toxicity, at elevated concentrations. 

### 4.3. Demographic and Clinical Data

Demographic, clinical, and laboratory data were collected through systematic review of electronic medical records using standardized data collection forms. The variables gathered included age, weight, body mass index (BMI), gender, Isavuconazole formulation and dosing regimen, treatment duration, and laboratory parameters related to liver function [alanine aminotransferase (ALT), aspartate aminotransferase (AST), gamma-glutamyl transferase (GGT), alkaline phosphatase (ALP), and total serum bilirubin].

Clinical outcomes, serious adverse events, breakthrough infections, and concomitant medications were also recorded at baseline and at each TDM episode. 

Additional clinical variables, such as the use of continuous renal replacement therapy (CRRT), were included to explore potential correlations with IsaPlasm. 

Possible adverse events, including QT interval prolongation, neutropenia, hepatotoxicity, headache, abdominal pain, nausea, and vomiting, were documented through daily clinical assessments. Potential drug–drug interactions were assessed using the Lexicomp^®^ Drug Interactions database (Wolters Kluwer Health) [34].

### 4.4. Blood Sample Collection and Analysis

The day of first Isavuconazole administration was designated as day 0. IsaPlasm was measured 4–5 days after treatment initiation to optimize dosing in terms of efficacy, toxicity, and resistance prevention. 

Blood samples were collected from a peripherally inserted radial catheter (arterial line) at specific time points relative to intravenous drug administration: 0 h (trough concentration or Minimum Plasma Concentration (Cmin))d 1 h (peak concentration or Maximum Plasma Concentration (Cmax)), 2, 4, 6, and 12 h post-infusion. Additionally, blood samples were obtained at 0 and 1 h from both pre-oxygenator and post-oxygenator sites of the ECMO circuit to assess drug sequestration within the circuit. The percentage of drug loss was calculated as:

Sequestration (%) = [(IsaPlasm post-oxygenator circuit) − (IsaPlasm pre-oxygenator circuit)]/(IsaPlasm post-oxygenator circuit).

Differences greater than 10% at these time points were considered indicative of possible drug loss due to retention in the ECMO circuit. 

Plasma Isavuconazole concentrations were measured using high-performance liquid chromatography (HPLC) coupled with a fluorescent detector (Shimadzu Nexera RF-20Axs detector, Kyoto, Japan). Routine internal quality controls were included in each analytical batch, and external quality control assessments were performed bimonthly. The lower limit of quantification was 0.25 µg/mL, with acceptable intra- and inter-assay variability set at ≤10%.

### 4.5. Pharmacokinetics Analysis

PK parameters were estimated through non-compartmental analysis. Key PK parameters included terminal half-life (t1/2), peak concentration (Cmax), time to reach Cmax (Tmax), apparent volume of distribution (Vd), clearance (CL), and area under the concentration–time curve (AUC_0–12_ or AUC_0–24_). The elimination rate constant (k) was determined from the terminal slope of the log-transformed concentration–time curve. Clearance (CL) was calculated as the administered dose divided by AUC, and volume of distribution (Vd) was calculated as dose divided by (k·AUC). 

ISA AUC_0–12_ or AUC_0–24_ was estimated using the log-linear trapezoidal method with a pre-formatted spreadsheet from the pharmacokinetics department of our centre. To enable a meaningful comparison of systemic exposure across different dosing regimens, AUC values obtained from patients receiving Isavuconazole 200 mg every 12 h were multiplied by a factor of two to estimate the total daily AUC (AUC_0–24_). This adjustment allowed comparison with AUC values from patients who received the drug once daily (200 mg every 24 h). The AUC values reported in the Results section represent standardized daily exposures (AUC_0–24_). This extrapolation approach is consistent with previously published pharmacokinetic analyses of Isavuconazole, which estimated 24 h AUC by extrapolating from shorter sampling intervals using individual elimination constants, under the assumption of linear pharmacokinetics and steady-state conditions [35].

### 4.6. Extracorporeal Circuit

The indications for ECMO support, circuit modality, and adjustments were determined by the clinical care team. Patients received either a CardioHelp™ (Maquet Cardiovascular, Rastatt, Germany) or Xenios AG™ (Fresenius Medical Care company, Bad Homburg vor der Höhe, Germany) centrifugal pump system. Blood flow was initiated at 1000 mL/min and titrated upwards every 5 min to a maximum of 80 mL/kg/min or 7 L/min, according to patient needs. The oxygenator used was integrated into the CardioHelp™ or Xenios AG™ system, so the gas flow was adjusted based on the patient’s PaCO_2_. Both devices incorporated polymethylpentene (PMP) gas exchange membranes with a surface area of 1.3 m^2^, impermeable to plasma. A heat exchanger was used to maintain normothermia (37 °C). All ECMO circuits were primed with normal saline prior to initiation. 

### 4.7. Statistical Analysis

Descriptive statistics were reported as means and standard deviations for normally distributed continuous variables, medians and interquartile ranges (IQR) for non-normally distributed variables. Categorical variables were presented as absolute and relative frequencies. Comparisons between groups were performed using Fisher’s exact test for categorical variables, paired *t*-test for normally distributed continuous variables, and the Wilcoxon signed-rank test for non-normally distributed continuous variables. A *p*-value below 0.05 was considered statistically significant. All statistical analyses were conducted using IBM SPSS Statistics 23 (SPSS, Inc., Chicago, IL, USA). 

## 5. Conclusions

This study provides insights into the pharmacokinetic behavior of Isavuconazole in critically ill patients receiving VV-ECMO support. Marked interindividual variability in drug exposure and evidence of circuit-related sequestration, particularly following recent ECMO system changes, underscore the need for individualized dosing and routine therapeutic drug monitoring. 

Moreover, our findings emphasize the importance of including ECMO-related pharmacokinetic considerations in future antifungal dosing guidelines to optimize patient outcomes. While standard dosing may be adequate for some patients, others may require dose adjustments to ensure optimal antifungal efficacy and safety. Further research in larger cohorts is warranted to establish clear exposure–response relationships and refine dosing recommendations in this complex population.

## Figures and Tables

**Table 1:** Patient characteristics, treatment data, and Isavuconazole exposure.

Patient ID	1	2	3	4	5
Sex	Male	Male	Female	Female	Male
Age (years)	65	61	54	44	64
Weight (kg)	84	65	67	60	111
BMI (kg/m^2^)	26.8	23.9	22.6	25.0	36.4
SOFA score	12	12	12	11	12
Outcomes	Died			Died	
Comorbidities					
Renal Failure (diálisis)	no	no	no	no	no
**Isavuconazole indication**	CAPA	CAPA	CAPA	CAPA	Empirical treatment for suspected fungal superinfection
**Laboratory parameters (on AUC day)**					
Serum Creatinine (mg/dL)	0.92	0.70	0.74	1.21	0.46
Bilirubin (mg/dL)	1.42	0.70	5.62	0.84	0.60
ALT (U/L)	116	38	41	27	42
AST (U/L)	129	31	141	114	37
Serum Albumin (g/dL)	2.4	4.1	2.4	3.6	2.8
**ECMO characteristics**					
Indication	ARDS	ARDS	ARDS	ARDS	ARDS
ECMO modality	V-V ECMO	V-V ECMO	V-V ECMO	V-V ECMO	VV
ECMO duration (days)	24	42	20	15	27
Circuit change (day)	22	20	3	3	2
**Pharmacokinetics**					
ECMO circuit age at PK sampling (days)	22	20	3	3	2
Isavuconazole IV dosage	200 mg/BID	200 mg/BID	200 mg/QD	200 mg/QD	200 mg/QD
AUC (µg·h/mL) 0–12	125.2	144.3	-	-	-
AUC (µg·h/mL) 0–24	-	-	227.3	137.5	395.4
Cmin (mcg/mL)	2.00	2.16	3.96	2.32	1.13
T1/2 (h)	33.01	34.66	31.51	21.66	138.63
Kel	0.021	0.020	0.022	0.032	0.05
Vd (L/kg)	623	630	150	311	598
Cl (L/h)	1.60	1.39	0.88	1.45	0.51

CAPA: COVID-19-associated pulmonary aspergillosis. ALT: alanine aminotransferase. AST: aspartate aminotransferase. ECMO: extracorporeal membrane oxygenation. V-V ECMO: veno-venous ECMO. AUC: area under the concentration–time curve. Cmin: minimum plasma concentration. T1/2: elimination half-life. Kel: elimination rate constant. Vd: volume of distribution. Cl: clearance.

**Table 2:** Trough (C_0_) and peak (C_1_) Isavuconazole concentrations measured at three ECMO circuit sites (pre-oxygenator, post-oxygenator, and arterial), and percentage differences between pre- and post-oxygenator samples.

Patient	C_0_ Pre-ox (mg/L)	C_0_ Post-ox (mg/L)	C_0_ Arterial (mg/L)	ΔC_0_ (%)	C_1_ Pre-ox (mg/L)	C_1_ Post-ox (mg/L)	C_1_ Arterial (mg/L)	ΔC_1_ (%)
**P1**	1.95	1.86	2.00	−5.0	3.34	3.21	3.20	−4.05
**P2**	2.17	2.09	2.16	−4.0	2.99	2.96	3.17	−1.01
**P3**	4.94	3.18	3.96	−55.0	13.2	15.1	13.3	12.58
**P4**	2.43	2.34	2.32	−4.0	5.74	6.11	6.42	6.06
**P5**	2.38	1.62	1.13	−47.0	4.43	3.65	3.34	−21.37

Δ (%) = %sequestration = ((Postox − Preox)/Postox) × 100.

**Table 3:** Pharmacokinetic parameters of Isavuconazole in ECMO patients compared to published data in non-ECMO settings.

PK Parameter	Present Cases (ECMO) Median (IQR) 200 mg q/12 h	Present Cases (ECMO) Median (IQR) 200 mg q/24 h	Invasive Fungal Disease [17] (Non-ECMO) Mean ± SD	Prescribing Info: [18] Mean ± SD
AUC (μg·h/mL)	227.3 (182.4–311.4)	125.2 (81–185.8)	87.1 ± 41	73.2 ± 12.4
Cmin (μg/mL)	2.0 (1.7–3.1)	2.4 (1.8–3.2)	–	–
t1/2 (h)	33 (22.7–33.1)	31.5 (26.6–85.1)	80.4 ± 33	–
kel (1/h)	0.021 (0.021–0.023)	0.022 (0.014–0.027)	–	–
Vd (L)	761 (727–832)	454 (427–733)	361.2 ± 166.3	304 ± 86.6
CL (L/h)	1.6 (1.5–3.4)	0.9 (0.7–1.2)	2.5 ± 1.6	2.8 ± 0.52

AUC: area under the curve. Cmin: minimum concentration. t½: half-life. Kel: elimination rate constant. Vd: volume of distribution. CL: clearance. Note: Values from different studies may not be directly comparable due to variations in population characteristics, disease severity, dosing regimens, and PK sampling methodologies.

## Data Availability

All data relevant to the case report are included in the manuscript. Additional data are available from the authors upon reasonable request.

## Abbreviations

The following abbreviations are used in this manuscript:

AUC	Area Under the Curve
Cmin	Minimum Plasma Concentration
Cmax	Maximum Plasma Concentration
T1/2	Elimination Half-Life
kel	Elimination Rate Constant
Vd	Volume of Distribution
CL	Clearance
ECMO	Extracorporeal Membrane Oxygenation
ARDS	Acute Respiratory Distress Syndrome
TDM	Therapeutic Drug Monitoring
PPB	Plasma Protein Binding
MOD	Multiple Organ Dysfunction
CRRT	Continuous Renal Replacement Therapy
CAPA	COVID-19-Associated Pulmonary Aspergillosis
V-V ECMO	Veno-Venous ECMO
BMI	Body Mass Index
AST	Aspartate Aminotransferase
ALT	Alanine Aminotransferase
ALP	Alkaline Phosphatase
GGT	Gamma-Glutamyl Transferase
ISA	Isavuconazole
HPLC	High-Performance Liquid Chromatography
CRRT	Continuous Renal Replacement Therapy
PaCO_2_	Partial Pressure of Carbon Dioxide
SD	Standard Deviation
IQR	Interquartile Range
SIRS	Systemic Inflammatory Response Syndrome
QT	QT interval of the electrocardiogram

## References

[1] Ellsworth M., Ostrosky-Zeichner L. (2020). Isavuconazole: Mechanism of Action, Clinical Efficacy, and Resistance. J. Fungi.

[2] McCarthy M.W., Moriyama B., Petraitiene R., Walsh T.J., Petraitis V. (2018). Clinical Pharmacokinetics and Pharmacodynamics of Isavuconazole. Clin. Pharmacokinet..

[3] Van Daele R., Debaveye Y., Vos R., Van Bleyenbergh P., Brüggemann R.J., Dreesen E., Elkayal O., Guchelaar H.J., Vermeersch P., Lagrou K. (2021). Concomitant Use of Isavuconazole and CYP3A4/5 Inducers: Where Pharmacogenetics Meets Pharmacokinetics. Mycoses.

[4] Gaffney S., Kelly D.M., Rameli P.M., Kelleher E., Martin-Loeches I. (2023). Invasive Pulmonary Aspergillosis in the Intensive Care Unit: Current Challenges and Best Practices. APMIS.

[5] Millar J.E., Fanning J.P., McDonald C.I., McAuley D.F., Fraser J.F. (2016). The Inflammatory Response to Extracorporeal Membrane Oxygenation (ECMO): A Review of the Pathophysiology. Crit. Care.

[6] Koehler P., Bassetti M., Chakrabarti A., Chen S.C.A., Colombo A.L., Hoenigl M., Klimko N., Lass-Flörl C., Oladele R.O., Vinh D.C. (2021). Defining and Managing COVID-19-Associated Pulmonary Aspergillosis: The 2020 ECMM/ISHAM Consensus Criteria for Research and Clinical Guidance. Lancet Infect. Dis..

[7] Andes D., Kovanda L., Desai A., Kitt T., Zhao M., Walsh T.J. (2018). Isavuconazole Concentration in Real-World Practice: Consistency With Results From Clinical Trials. Antimicrob. Agents Chemother..

[8] Mikulska M., Melchio M., Signori A., Ullah N., Miletich F., Sepulcri C., Limongelli A., Giacobbe D.R., Balletto E., Russo C. (2024). Lower Blood Levels of Isavuconazole in Critically Ill Patients Compared with Other Populations: Possible Need for Therapeutic Drug Monitoring. J. Antimicrob. Chemother..

[9] Lyster H., Shekar K., Watt K., Reed A., Roberts J.A., Abdul-Aziz M.-H. (2023). Antifungal Dosing in Critically Ill Patients on Extracorporeal Membrane Oxygenation. Clin. Pharmacokinet..

[10] Pau-Parra A., Sosa Garay M., Doménech Moral L., Díez Poch M., Martínez Pla M., Gallart E., Vima Bofarull J., Nuvials X., García-García S., Doménech Vila J.M. (2025). Therapeutic Drug Monitoring-Guided High-Dose Isavuconazole Therapy for Invasive Pulmonary Aspergillosis in a Patient on Extracorporeal Membrane Oxygenation Support. J. Chemother..

[11] Höhl R., Bertram R., Kinzig M., Haarmeyer G.-S., Baumgärtel M., Geise A., Muschner D., Prosch D., Reger M., Naumann H.-T. (2022). Isavuconazole Therapeutic Drug Monitoring in Critically Ill ICU Patients: A Monocentric Retrospective Analysis. Mycoses.

[12] Perez L., Corne P., Pasquier G., Konecki C., Sadek M., Le Bihan C., Klouche K., Mathieu O., Reynes J., Cazaubon Y. (2023). Population Pharmacokinetics of Isavuconazole in Critical Care Patients with COVID-19-Associated Pulmonary Aspergillosis and Monte Carlo Simulations of High Off-Label Doses. J. Fungi.

[13] Furfaro E., Signori A., Di Grazia C., Dominietto A., Raiola A.M., Aquino S., Ghiggi C., Ghiso A., Ungaro R., Angelucci E. (2019). Serial Monitoring of Isavuconazole Blood Levels during Prolonged Antifungal Therapy. J. Antimicrob. Chemother..

[14] Lamoth F., Glampedakis E., Boillat-Blanco N., Oddo M., Pagani J.-L. (2020). Incidence of Invasive Pulmonary Aspergillosis among Critically Ill COVID-19 Patients. Clin. Microbiol. Infect..

[15] Riera J., Barbeta E., Tormos A., Mellado-Artigas R., Ceccato A., Motos A., Fernández-Barat L., Ferrer R., García-Gasulla D., Peñuelas O. (2023). Effects of Intubation Timing in Patients with COVID-19 throughout the Four Waves of the Pandemic: A Matched Analysis. Eur. Respir. J..

[16] Patel J.S., Kooda K., Igneri L.A. (2023). A Narrative Review of the Impact of Extracorporeal Membrane Oxygenation on the Pharmacokinetics and Pharmacodynamics of Critical Care Therapies. Ann. Pharmacother..

[17] Kovanda L.L., Desai A.V., Lu Q., Townsend R.W., Akhtar S., Bonate P., Hope W.W. (2016). Isavuconazole Population Pharmacokinetic Analysis Using Nonparametric Estimation in Patients with Invasive Fungal Disease (Results from the VITAL Study). Antimicrob. Agents Chemother..

[18] Cresemba® (Isavuconazole) (2016). Annex I of Summary of Product Characteristics.

[19] Shekar K., Abdul-Aziz M.H., Cheng V., Burrows F., Buscher H., Cho Y.-J., Corley A., Diehl A., Gilder E., Jakob S.M. (2023). Antimicrobial Exposures in Critically Ill Patients Receiving Extracorporeal Membrane Oxygenation. Am. J. Respir. Crit. Care Med..

[20] Miller M., Kludjian G., Mohrien K., Morita K. (2022). Decreased Isavuconazole Trough Concentrations in the Treatment of Invasive Aspergillosis in an Adult Patient Receiving Extracorporeal Membrane Oxygenation Support. Am. J. Health-Syst. Pharm..

[21] Mendoza-Palomar N., Melendo-Pérez S., Balcells J., Izquierdo-Blasco J., Martín-Gómez M.T., Velasco-Nuño M., Rivière J.G., Soler-Palacin P. (2021). Influenza-Associated Disseminated Aspergillosis in a 9-Year-Old Girl Requiring ECMO Support. J. Fungi.

[22] Zhao Y., Seelhammer T.G., Barreto E.F., Wilson J.W. (2020). Altered Pharmacokinetics and Dosing of Liposomal Amphotericin B and Isavuconazole During Extracorporeal Membrane Oxygenation. Pharmacotherapy.

[23] Kriegl L., Hatzl S., Zurl C., Reisinger A.C., Schilcher G., Eller P., Gringschl Y., Muhr T., Meinitzer A., Prattes J. (2022). Isavuconazole Plasma Concentrations in Critically Ill Patients during Extracorporeal Membrane Oxygenation. J. Antimicrob. Chemother..

[24] Zurl C., Waller M., Schwameis F., Muhr T., Bauer N., Zollner-Schwetz I., Valentin T., Meinitzer A., Ullrich E., Wunsch S. (2020). Isavuconazole Treatment in a Mixed Patient Cohort with Invasive Fungal Infections: Outcome, Tolerability and Clinical Implications of Isavuconazole Plasma Concentrations. J. Fungi.

[25] Kovanda L.L., Marty F.M., Maertens J., Desai A.V., Lademacher C., Engelhardt M., Lu Q., Hope W.W. (2017). Impact of Mucositis on Absorption and Systemic Drug Exposure of Isavuconazole. Antimicrob. Agents Chemother..

[26] Schmitt-Hoffmann A., Roos B., Spickermann J., Heep M., Peterfaí E., Edwards D.J., Stoeckel K. (2009). Effect of Mild and Moderate Liver Disease on the Pharmacokinetics of Isavuconazole after Intravenous and Oral Administration of a Single Dose of the Prodrug BAL8557. Antimicrob. Agents Chemother..

[27] Desai A., Schmitt-Hoffmann A.-H., Mujais S., Townsend R. (2016). Population Pharmacokinetics of Isavuconazole in Subjects with Mild or Moderate Hepatic Impairment. Antimicrob. Agents Chemother..

[28] Bolcato L., Thiebaut-Bertrand A., Stanke-Labesque F., Gautier-Veyret E. (2022). Variability of Isavuconazole Trough Concentrations during Longitudinal Therapeutic Drug Monitoring. J. Clin. Med..

[29] Desai A.V., Kovanda L.L., Hope W.W., Andes D., Mouton J.W., Kowalski D.L., Townsend R.W., Mujais S., Bonate P.L. (2017). Exposure-Response Relationships for Isavuconazole in Patients with Invasive Aspergillosis and Other Filamentous Fungi. Antimicrob. Agents Chemother..

[30] Bertram R., Naumann H., Bartsch V., Hitzl W., Kinzig M., Haarmeyer G., Baumgärtel M., Geise A., Muschner D., Nentwich J. (2023). Clinical and Demographic Factors Affecting Trough Levels of Isavuconazole in Critically Ill Patients with or without COVID-19. Mycoses.

[31] Zhou J., Xu B., Zheng Y., Huang H., Wei Z., Chen S., Huang W., Liu M., Zhang Y., Wu X. (2024). Optimization of Oral Isavuconazole Dose for Population in Special Physiological or Pathological State: A Physiologically Based Pharmacokinetics Model-Informed Precision Dosing. J. Antimicrob. Chemother..

[32] Hatzl S., Kriegl L., Posch F., Schilcher G., Eller P., Reisinger A., Grinschgl Y., Muhr T., Meinitzer A., Hoenigl M. (2023). Early Attainment of Isavuconazole Target Concentration Using an Increased Loading Dose in Critically Ill Patients with Extracorporeal Membrane Oxygenation. J. Antimicrob. Chemother..

[33] Jansen A.M.E., Mertens B., Spriet I., Verweij P.E., Schouten J., Wauters J., Debaveye Y., ter Heine R., Brüggemann R.J.M. (2023). Population Pharmacokinetics of Total and Unbound Isavuconazole in Critically Ill Patients: Implications for Adaptive Dosing Strategies. Clin. Pharmacokinet..

[34] Lexicomp (1978). Lexi-Interact Online.

[35] Bergmann F., Wölfl-Duchek M., Jorda A., Al Jalali V., Leutzendorff A., Sanz-Codina M., Gompelmann D., Trimmel K., Weber M., Eberl S. (2024). Pharmacokinetics of Isavuconazole at Different Target Sites in Healthy Volunteers after Single and Multiple Intravenous Infusions. J. Antimicrob. Chemother..

